# Bioenergetic myths of energy transduction in eukaryotic cells

**DOI:** 10.3389/fmolb.2024.1402910

**Published:** 2024-06-17

**Authors:** Guy C. Brown

**Affiliations:** Department of Biochemistry, University of Cambridge, Cambridge, United Kingdom

**Keywords:** cell metabolism, mitochondria, glycolysis, cancer, energetics, Warburg effect, oxidative stress, bioenergetics

## Abstract

The study of energy transduction in eukaryotic cells has been divided between Bioenergetics and Physiology, reflecting and contributing to a variety of Bioenergetic myths considered here: 1) ATP production = energy production, 2) energy transduction is confined to mitochondria (plus glycolysis and chloroplasts), 3) mitochondria only produce heat when required, 4) glycolysis is inefficient compared to mitochondria, and 5) mitochondria are the main source of reactive oxygen species (ROS) in cells. These myths constitute a ‘mitocentric’ view of the cell that is wrong or unbalanced. In reality, mitochondria are the main site of energy dissipation and heat production in cells, and this is an essential function of mitochondria in mammals. Energy transduction and ROS production occur throughout the cell, particularly the cytosol and plasma membrane, and all cell membranes act as two-dimensional energy conduits. Glycolysis is efficient, and produces less heat per ATP than mitochondria, which might explain its increased use in muscle and cancer cells.

## Introduction

In principle, Bioenergetics is the study of the energetic aspects of biological processes, and the flow of energy through cells and organisms. However, in practise, Bioenergetics has largely confined itself to studying the energy metabolism of mitochondria, chloroplasts and bacteria (see, for example, the contents of Bioenergetics journals or conferences or the classic Bioenergetics textbook: Nicholls and Ferguson, 2013). This may, in part, have arisen from equating ATP production with energy production, and ATP use with energy use, related to the idea that ATP is the energy currency of the cell (Lipmann, 1941; Krebs and Kornberg, 1957). However, disciplinary boundaries helped confine Bioenergetics largely to ATP production, while Physiology considered the plasma membrane and muscle contraction as its territory, and other ATP use was left over for Biochemistry. Of course, there are many honourable exceptions of Physiologists and Bioenergeticists considering the whole cell (e.g., Nicholls, 2016; Yang et al., 2021; Barclay and Curtin, 2023), but generations of Bioenergeticists have been led to believe that mitochondria are all there is to energy transduction in cells. And this has resulted in a ‘mitocentric’ view of the cell, i.e., the view that mitochondria are central to cellular processes, in particular energy transduction, including the view that mitochondria produce the cells energy, while the rest of the cell consumes that energy. This is a flawed concept of energy transduction in eukaryotic cells, and some of the Bioenergetic myths that constitute this view are pointed out here, hopefully enabling a more balanced and unified view of cellular energy transduction.

## Bioenergetic myth 1. Mitochondrial ATP production = energy production

ATP production (in particular, mitochondrial ATP production) has sometimes been equated with energy production (where ‘energy’ can refer to total energy, free energy or internal energy). However, this is clearly incorrect because: i) the first law of thermodynamics tells us that total energy is conserved in all processes, ii) the second law of thermodynamics tells us that free energy is dissipated in all processes, and iii) mitochondrial ATP production dissipates about 80% of the internal energy it receives, and produces about 75% of all cellular heat (Supplementary Appendix S1). This can be calculated from the enthalpy change (ΔH) of mitochondrial ATP synthesis dived by ΔH of ATP synthesis plus ATP hydrolysis, i.e., the ΔH of substrate oxidation. The latter ΔH is between −217 and −235 kJ/mol O for oxidation of glucose, lactate or fatty acids (Gnaiger and Kemp, 1990), whereas ΔH of ATP hydrolysis is −20 kJ/mol ATP, so if the effective P/O ratio in cells is 2, then -ΔH of mitochondrial ATP synthesis is 226–40 = 186 kJ/mol O (Prusiner and Poe, 1968; Rolfe and Brown, 1997). Thus, about 80% of the internal energy received by mitochondria (ΔH of substrate oxidation) is dissipated as heat (-ΔH of mitochondrial ATP synthesis). The other 20% of the ΔH of substrate oxidation is dissipated as heat by ATP usage and subsequent processes in the rest of the cell. Heat is also produced (and absorbed) elsewhere in metabolism, but most of this can be accounted for by the 10% of cellular oxygen consumption that is not mitochondrial, so about 75% of the heat produced by cellular metabolism is produced by mitochondria (Prusiner and Poe, 1968; Rolfe and Brown, 1997). Note that the effective P/O ratio is the actual ratio of mitochondrial ATP production to oxygen consumption in cells, which is lower than the maximal, theoretical P/O ratio largely because of mitochondrial proton leak (Rolfe and Brown, 1997). Thus, ATP production i) does not change the total amount of energy, ii) dissipates free energy, and iii) dissipates most of the internal energy it receives.

**FIGURE 1:**
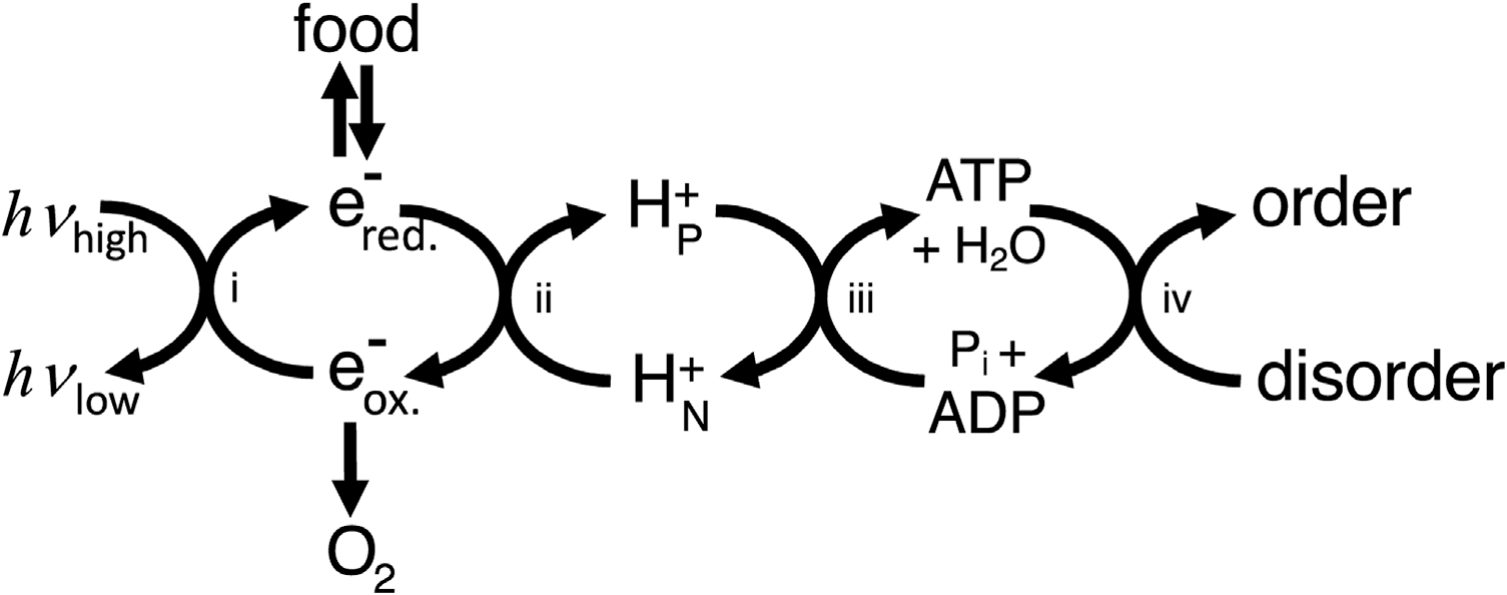
Cellular energy transduction consists mainly of a chain of coupling cycles that couple the ΔG-requiring ordering process required for growth and maintenance of the cell to sources of ΔG in the environment (including light for photosynthetic organisms and food plus oxygen for animals). i) Photosynthesis couples light energy, absorbed at optical frequencies (*hv*_high_) and emitted at infrared frequencies (*hv*_low_), to moving electrons from oxidising (e^-^_ox._) to reducing (e^-^_red._) molecules. ii) Electron transport chains couple electron transport from reduced to oxidised molecules to proton transport across a membrane from electrically N (negative) to P (positive) compartments of the cell. iii) The ATP synthase couples proton return to ATP synthesis. iv) ATP use couples ATP hydrolysis to the ordering process of the cell (including transport, biosynthesis and signalling). Note that this way of depicting energy transduction downplays coupling cycles within ATP usage and ignores other coupling chains, for example, via NADPH.

In what sense then is ATP production providing energy for the cell? To answer this, we can use the concept of energy coupling i.e., coupling enzymes or transporters couple processes with a positive free energy change to reactions with a negative free energy change (e.g., ATP hydrolysis), and as long as the net free energy change is negative then the coupled process will go forward (Supplementary Appendix S1). This coupling is energy transduction, i.e., the transfer of free energy from one mode/molecule to another. Thus, ATP production provides energy to the cell in the sense that it provides a readily useable source of free energy (ATP), the hydrolysis of which can be coupled by proteins to free energy-requiring processes. However, the ATP needs to be regenerated using a chain of coupling cycles, via: i) light-induced reduction of substrates in photosynthetic organisms, and/or ii) oxidation of reduced substrates coupled to proton transport, and iii) protons return coupled to ATP synthesis (Figure 1). Because energy transduction pathways largely consist of coupling chains, the architecture of energy transducing pathways differs fundamentally from metabolic pathways and signal transduction pathways (Supplementary Appendix S2).

**FIGURE 2:**
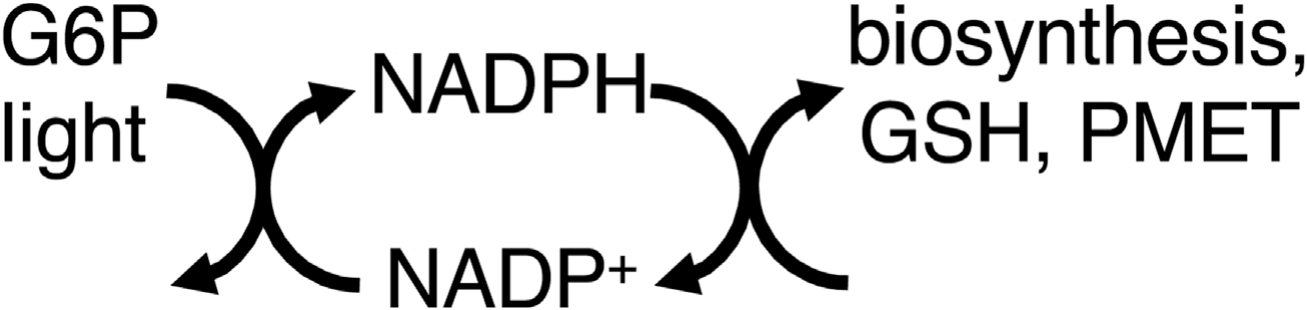
NADPH couples light (via photosynthesis) and G6P (glucose-6-phosphate, via the pentose phosphate pathway) to biosynthesis, GSH (glutathione) reduction, and PMET (plasma membrane electron transport).

Thus, mitochondria provide energy to the cell by coupling food oxidation to ATP synthesis, which transfers most of the free energy in the former to the latter. However, equating ATP production with energy production leads to neglect of other pathways of energy transduction, such as via NADPH (Chandel, 2021). NADPH is produced by: i) ferredoxin–NADP^+^ reductase as part of photosynthesis, ii) glucose-6-phosphate dehydrogenase (G6PDH) of the pentose phosphate pathway, and iii) mitochondrial dehydrogenases and transhydrogenase (Chandel, 2021). NADPH is used for: synthesis of fatty acids, nucleic acids, cholesterol, steroids, ascorbate, xylitol, and photosynthetic carbon fixation. NADPH is regarded as a source of reducing equivalents for these synthetic pathways, but not necessarily a source of free energy (Chandel, 2021), partly because reduction by NAD(P)H combines the characteristics of both metabolic and energy transduction pathways in that both matter and energy are transferred (Supplementary Appendix S2). However, many energy coupling processes consist of sub-steps/reactions in which both matter and energy are transferred, e.g., electron transport chains (Supplementary Appendix S3) or P-type ATPases. Where the NADPH-driven synthetic reactions are reversed by catabolism coupled to production of NADH (e.g., by fatty acid or sugar oxidation), the NADPH-driven synthetic reactions can be regarded as storage of free energy, and therefore part of energy transduction. NADPH also reduces glutathione, which goes on to reduce protein thiols and hydrogen peroxide, displacing these reactions far from equilibrium (Figure 2).

**FIGURE 3:**
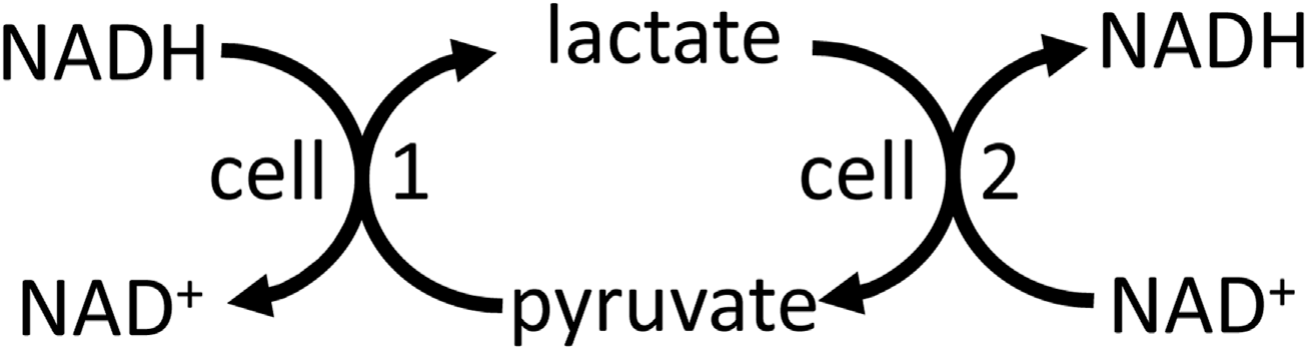
Reversal of the lactate dehydrogenase reaction, plus transport of lactate and pyruvate, in different cells and cellular compartments may transfer redox energy.

NADH/NAD^+^ is well known to mediate energy transduction via coupling the oxidation of carbohydrate, fats and amino acids to the mitochondrial respiratory chain (Supplementary Appendix S3). And NADH-linked dehydrogenases in different compartments, cells and tissues can be used to shuttle energy between these (Brooks et al., 2022). For example, lactate dehydrogenase in peroxisomes can use NADH from fatty acid oxidation to reduce pyruvate to lactate, which is then transported to the cytosol where lactate dehydrogenase converts the lactate back to pyruvate, producing cytosolic NADH (McClelland & 2003). Cytosolic NADH can be shuttled into the mitochondria via reversal of malate dehydrogenase in cytosol and mitochondria by the malate-aspartate shuttle (Brooks et al., 2022). Cytosolic NADH can be shuttled to neighbouring cells (e.g., between glial cells and neurons in the brain), or cells at the other end of the body, via reversal of lactate dehydrogenase in the respective cells, and this can in principle act to shuttle energy between cells (Figure 3). Note that this differs from the lactate shuttle (that shuttles lactate between lactate producing and consuming compartments or cells), in that lactate and pyruvate are only interconverted with no net production or use, but both shuttles might operate together or independently. Lactate to pyruvate exchange and transport rates are known to be much faster than net lactate production and consumption (Romijn et al., 1994; Khegai et al., 2014), so if the latter contributes to energy transfer between cells (via the lactate shuttle), then the former should also contribute. Thus, NADH and NADPH are both part of energy transduction.

It is interesting to note that the mitochondrial respiratory chain is normally conceived of as an electron transport chain with electrons starting at complex I or complex II, and proceeding via complex III to complex IV, and ending at oxygen reduction; whereas in fact the chain consists of coupled cycles, which can be considered to start with oxygen at complex IV, and there are bioenergetic reasons for preferring this reverse ordering (Supplementary Appendix S3).

Electron transport occurs across the plasma membrane, coupling NAD(P)H oxidation in the cytosol to the extracellular reduction of oxygen to O_2_^−^, H_2_O_2_ or H_2_O, or reduction of extracellular thiols via membrane ubiquinone (Del Principe et al., 2011; Morré and Morré, 2012). This may function in part to transfer/transduce free energy to the extracellular space. It is still somewhat mysterious what powers the extracellular space (if it is powered at all), which is strange for such a large space with many important functions. The cytosol and intra-organelle/vesicle compartments are powered mainly by NAD(P)H and ATP (and other nucleotides), while the membranes are mainly powered by proton or sodium electrochemical gradients, but whether or what powers the extracellular space is unclear. However, many extracellular proteins are regulated by oxidation/reduction, particularly of protein thiols, catalysed by extracellular thiol oxidoreductases, powered by reductants or oxidants generated by trans-plasma membrane electron transport (Lorenzen et al., 2020; Tanaka et al., 2020).

In conclusion, mitochondrial ATP production is not equivalent to energy production, because mitochondria do not and can not produce energy, rather they are part of a chain of coupling cycles that supply free energy throughout the cell, and there are other coupling chains that do not involve mitochondria.

## Bioenergetic myth 2. ATP use = energy consumption

ATP usage has sometimes been equated with energy consumption (Lipmann, 1941; Krebs and Kornberg, 1957). It is true that ATP usage by cells is accompanied by loss of free energy and internal energy (as occurs in ATP production), but the energy is not all dissipated, but rather used for energy coupling. All ATP use by cells is coupled to some other reaction/process, usually an endergonic reaction. Thus, ATP use ≠ energy consumption, but rather energy conservation, and is part of the coupling chain that constitutes energy transduction in cells. So, it is a myth that energy transduction stops at ATP, indeed it could be argued that ATP use is the main energy transduction step in cells, because there are a vast range of ATP coupled reactions (>500) that power most processes in the cell (Manning et al., 2002). Thus, it is somewhat odd that Bioenergetics does not study ATP use, considering that it is one of the most important energy transduction processes in the cell.

ATP is at the beginning of a variety of energy transduction chains in the cytosol that funnel free energy to hundreds of processes throughout the cell. Creatine kinase and adenylate kinase use ATP to phosphorylate creatine and AMP respectively to enable storage and transport of phosphorylation free energy in cells, as part of these cytosolic energy transduction chains (Bessman and Carpenter, 1985; Savabi, 1994). Nucleoside-diphosphate kinase transfers the free energy of ATP to GTP, CTP and UTP, which then (directly or via further energy transduction chains) power protein synthesis, gluconeogenesis and G-proteins (GTP), lipid synthesis and protein glycosylation (CTP) and carbohydrate synthesis (UTP) respectively, as well as RNA synthesis (ATP, GTP, CTP & UTP) and many other processes (Nelson and Cox, 2021). The deoxy versions of these nucleotides (with TTP replacing UTP) power DNA synthesis.

There are over 500 protein kinases identified in the human genome that use ATP to phosphorylate one or more proteins, and about 30% of proteins are phosphorylated at any one time, many at multiple sites (Manning et al., 2002). This phosphorylation provides the protein with a slug of energy and charge that can change its conformation, activity and/or interactions. Dephosphorylation by protein phosphatase, involves exergonic hydrolysis of the protein phosphate, and returns the protein to the original state, but the net effect of the kinase and phosphatase is ATP hydrolysis. The amount of free energy provided to the protein depends on the residue phosphorylated: the phosphorylation potential of serine is 10 kJ/mol, of tyrosine 13 kJ/mol, of threonine 32 kJ/mol, of aspartate 52 kJ/mol and of histidine 55 kJ/mol (Hunter, 2022; http://equilibrator.weizmann.ac.il/), although the phosphorylation potential of amino acid residues will depend on residue environment. The phosphorylation potential of ATP in the cytosol of eukaryotic cells is about 60 kJ/mol (Rolfe and Brown, 1997), enabling ATP to fully phosphorylate serine, threonine and tyrosine residues, thereby enabling protein kinases to switch the state of proteins independent of energetic conditions. Phosphohistidine acts as an enzyme intermediate in a number of energy transducing enzymes, enabling efficient energy transfer (Hunter, 2022). Similarly, phosphorylation of aspartate residues is central to the mechanism of P-type ATPases, such as the sodium, proton and calcium pumps (Berman, 2001), enabling these pumps to transduce the energy of ATP into ion gradients relatively efficiently.

The quantitatively most important ion transporting ATPases in animals is the sodium pump, which powers the plasma membrane (Skou and Esmann, 1992). The sodium pump couples ATP hydrolysis to transporting 3 sodium ions out and 2 potassium ions in. This net charge transfer, together with the subsequent return of potassium ions out of the cell via potassium channels, generates the plasma membrane potential (Skou and Esmann, 1992). And this membrane potential and/or the sodium gradient is used to power other ion transport on the same membrane, including: sodium-coupled co-transport of sugars, amino acids, nucleosides, organic anions, inorganic anions (bicarbonate, chloride, phosphate, sulphate), and neurotransmitters, plus sodium-coupled antiport of calcium and protons, then the consequent electrochemical gradient of protons is used for proton-coupled co-transport of monocarboxylates, peptides and vitamins (Pizzagalli et al., 2021). The membrane potential is also used to drive the passive uptake of cations, such as amines and metal ions, including Fe^2+^ and Cu^2+^. Thus, the sodium pump drives an energy transduction chain on the plasma membrane of animal cells that powers the uptake of the necessities of life. The ion transport directly and indirectly powered by the sodium pump also enables: i) osmotic control of the cell challenged by its high osmolyte content, ii) the pre- and post-synaptic potentials and action potentials that enable neuronal signalling, and iii) the action potentials controlling muscle contraction (Skou and Esmann, 1992). Thus, the plasma membrane is one of the main bioenergetic organelles of the animal cell, and about 30% free energy flux passes through the sodium pump in mammals (Kelly and McBride, 1990; Rolfe and Brown, 1997). The efficiency of energy transduction by the sodium pump (ΔG of Na^+^ and K^+^ transported divided by ΔG of ATP hydrolysis, corrected for reaction stoichiometries) has been estimated to be: 57% in neurons (Erecinska and Silver, 1989), 67% in glia (Erecinska and Silver, 1989), and 85% in heart cells (Daut, 1987). These efficiencies are reasonably high (Berman, 2001).

**FIGURE 4:**
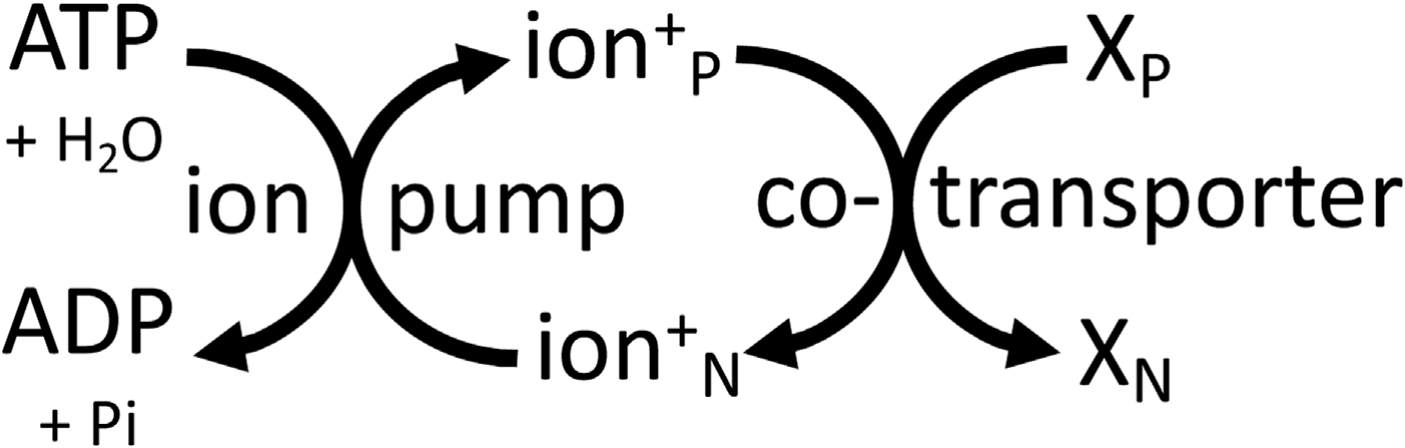
Cellular membranes are mainly powered by ATP-driven ion pumps (the sodium pump or proton pump) that generate delocalised, electrochemical gradients of ions (sodium or protons) across the membrane, which can be used by transporters elsewhere on the same membrane to power transport of other molecules (X) between the P (positive) and N (negative) sides of the membrane.

In plants, the sodium pump is replaced by a P-type proton pump of similar structure and mechanism, powered by ATP hydrolysis to generate an electrochemical proton gradient that powers transport of ions and substrates across the plasma membrane (Michalak et al., 2022). The plant vacuole is powered by V-type proton ATPases and by a pyrophosphatase-hydrolysis coupled proton pump (Harrison and Muench, 2018). The vacuole constitutes roughly 50% of plant cell volume and is the main store of substrates, the transport of which is directly or indirectly coupled to the electrochemical gradient of protons (Harrison and Muench, 2018). Intracellular vesicles of animals and plants (including endosomes, trans-Golgi, lysosomes, synaptic vesicles) are also powered by a V-type proton ATPase ((Martinoia et al., 2000; Harrison and Muench, 2018). Thus, all these membranes are powered by the electrochemical gradient of protons that directly or indirectly drives transport across these membranes. And the membrane acts as a two-dimensional power cable supplying a delocalised Δp that can drive any transporter that is plugged into this membrane. The same principle applies to the plasma membrane of animal cells, where the electrochemical gradient of sodium acts as a delocalised energy source anywhere on the cell surface, and therefore over large distances in neurons and muscle cells (Figure 4).

An important class of ATP user is the motor proteins, in particular the actinomyosin ATPase driving muscle contraction in animals, where ATP hydrolysis is coupled to movement. The contractile efficiency (mechanical work output divided by phosphorylation potential of ATP) has been estimated to be 20%–50% in a variety of muscles from mouse, rat, frog, dogfish and tortoise (Barclay and Curtin, 2023). Another quantitatively-important ATP use is protein synthesis, which uses about 30% of all ATP in mammalian cells (Kelly and McBride, 1990; Rolfe and Brown, 1997). This process is nominally inefficient as relatively little free energy is stored in the peptide bond, but clearly the correct amino acid sequence is crucial to life, and there may be a trade-off between energy use and accuracy of translation (Johansson & 2012).

In conclusion, cellular ATP use is not equivalent to energy consumption, but rather energy transduction. And energy transduction occurs throughout the cell, including via non-mitochondrial NADPH, NADH, ion gradients and electron transport.

## Bioenergetic Myth 3. Mitochondria only produce significant heat when needed

Mitochondria are often thought of as energy efficient and as producing minimal heat unless in specific conditions when heat is required. The thermodynamic efficiency of mitochondrial ATP synthesis (defined as ΔG of ATP hydrolysis divided by ΔG substrate oxidation, divided by the ratio of their rates) is about 65% in cellular conditions (Rolfe and Brown, 1997), which is reasonably efficient for a complex process. However, mitochondria dissipate (as heat) about 80% of the internal energy they receive, and are responsible for roughly 75% of the total heat production of mammalian cell (Prusiner and Poe, 1968; Rolfe and Brown, 1997). As heat production is essential to life in homeothermic animals, it follows that mitochondrial heat production is essential to human life and is an essential function of mitochondria in mammals and birds. The average surface temperature on earth is 15°C, average core body temperature of humans is 37°C, and humans become comatose at 32°C and die at 25–32°C of body temperature (Brown et al., 2012). If the efficiency of mitochondrial energy transduction was increased, then mitochondrial heat production would decrease, resulting in death. It would therefore seem likely that mitochondrial energy efficiency is limited in part by the need to produce heat in mammals and birds. It is well recognised that the mitochondrial proton leak makes mitochondria inefficient and heat producing, but this is only one of many contributions to mitochondrial inefficiency and heat production–almost all components of mitochondrial energy transduction contribute to heat production (Rolfe and Brown, 1997). Most heat production is within the inner mitochondrial membrane, which might help dissipate heat without protein denaturation (although there is no evidence for this). Heat is produced by non-mitochondrial metabolism and processes, but this is estimated to contribute only about 25% of total heat production in mammals (Rolfe and Brown, 1997).

Mitochondrial energy metabolism and core body temperature fall with human ageing and might contribute to reduced organ functions with age (Hernandes et al., 2021; Pontzer et al., 2021). However, brain temperature is two degrees higher that core temperature in humans and increases with age (Rzechorzek et al., 2022), which might impair brain function (Kim et al., 2022). Tissues with insufficient vascular removal of heat relative to mitochondrial heat production (such as exercising muscle, solid tumours, ischaemic tissues and aged brain) may reach excessive temperatures. Excessive temperatures may feed back to inhibit mitochondrial energy metabolism by heat-induced protein aggregation, which may help to limit excessive temperatures, but might also contribute to mitochondrial dysfunction in tumours or post-exercise muscle (Wilkening et al., 2018).

**FIGURE 5:**
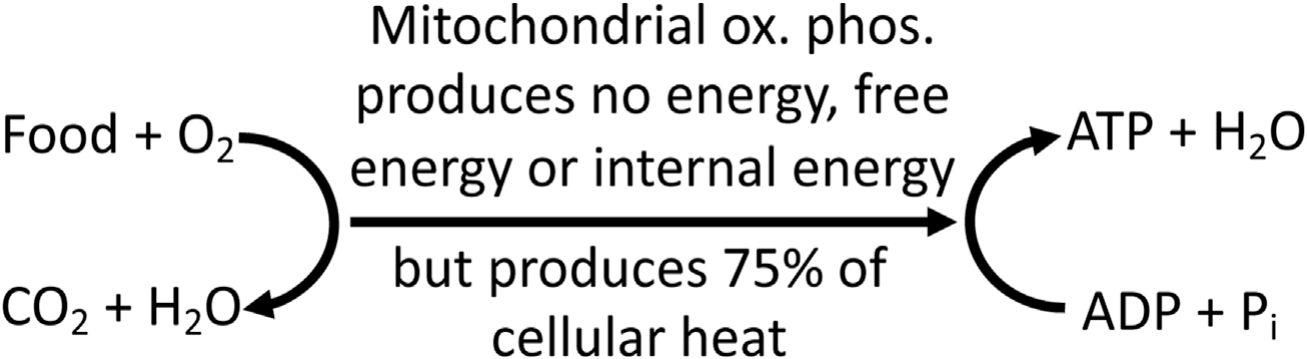
Mitochondrial oxidative phosphorylation (ox. phos.) couples food oxidation to ATP synthesis, but produces no energy, free energy or internal energy. It does, however, transfer free energy between these reactions, and produces most cellular and organism heat, which is essential to life in mammals and birds.

Thus, the idea that mitochondria only produce heat when needed is a myth: mitochondria produce most of cellular and organism heat, all of the time, including when resting, when exercising, when shivering and during non-shivering thermogenesis. And this heat production is an essential function of mitochondria in mammals and birds (Figure 5).

## Bioenergetic myth 4. Glycolysis is inefficient compared to mitochondria

Glycolysis is often said to be inefficient because it produces two molecules of ATP per molecule of glucose used, whereas the full oxidation of glucose by mitochondria can produce about 30 ATP per glucose molecule (Rich, 2003; Liberti and Locasale, 2016). However, this is a dubious comparison because glycolysis is the metabolism of glucose to pyruvate/lactate, whereas full oxidation of glucose is glycolysis of glucose to pyruvate/lactate plus mitochondrial oxidation of pyruvate to CO_2_. It does not really make sense to say that a small part of something is less efficient than the whole thing.

Thermodynamically, glycolysis is reasonably efficient because most the free energy of glucose metabolism to lactate is conserved in the phosphorylation of ADP to ATP. The ΔG of glucose → 2 lactate is about 200 kJ/mol glucose (http://equilibrator.weizmann.ac.il/), while the ΔG of ATP hydrolysis in the cytosol of liver, brain, muscle and heart has been estimated to be 60–65 kJ/mol ATP (Rolfe and Brown, 1997). So, the thermodynamic efficiency of glycolysis, defined as 2 x ΔG of ATP hydrolysis/ΔG of glucose → 2 lactate, is 125/200 = 61%, i.e., most of the free energy is conserved. The thermodynamic efficiency of mitochondrial oxidative phosphorylation is about 65% (Rolfe and Brown, 1997), so similar to that of glycolysis.

Interestingly, glycolysis produces less heat per ATP produced than mitochondrial ATP production. The enthalpy change of glucose oxidation is −469 kJ/mol per O_2_ consumed (Gnaiger and Kemp, 1990), and with an effective P/O ratio of 2 in cells (Rolfe and Brown, 1997), this is equivalent to heat production of 117 kJ/mol ATP produced. Mitochondrial oxidation of fat or protein have similar enthalpy changes (−442 kJ/mol O_2_) (Gnaiger and Kemp, 1990). The enthalpy change of glycolysis from glucose to lactate is between −63 and −70 kJ/mol lactate produced (Minikami S & de Verdier, 1976; Gnaiger and Kemp, 1990), and with an ATP/lactate ratio of 1, this is equivalent to heat production of 66 kJ/mol ATP produced. Thus, glycolysis produces roughly half (56%) as much heat per ATP produced as mitochondrial ATP production.

Cancer cells in solid tumours mainly use aerobic glycolysis to generate ATP, despite normally having sufficient oxygen and functional mitochondria (Zheng, 2012; Liberti and Locasale, 2016). A number of explanations have been suggested for this, none of which are entirely satisfactory (Zheng, 2012; Liberti and Locasale, 2016). However, the reduced heat production per ATP synthesised may be important in solid tumours with limited perfusion, and therefore in danger of overheating (Repasky et al., 2013). Solid tumours are generally poorly vascularised, and therefore hot, because blood flow is the only means of removing metabolic heat from internal tissues. Tumours are also sensitive to excess heat (Mallory et al., 2016), so there may be a selection pressure during tumorigenesis to switch to glycolysis to reduce heat production per ATP. Thus, this may explain the well-known finding that cancer cells switch to glycolysis during tumorigenesis (Liberti and Locasale, 2016). Similarly, maximally exercising skeletal muscle is known to be hot and sensitive to heat (Kenny et al., 2003), and therefore may benefit by switching to glycolysis for ATP production to reduce heat production.

**FIGURE 6:**
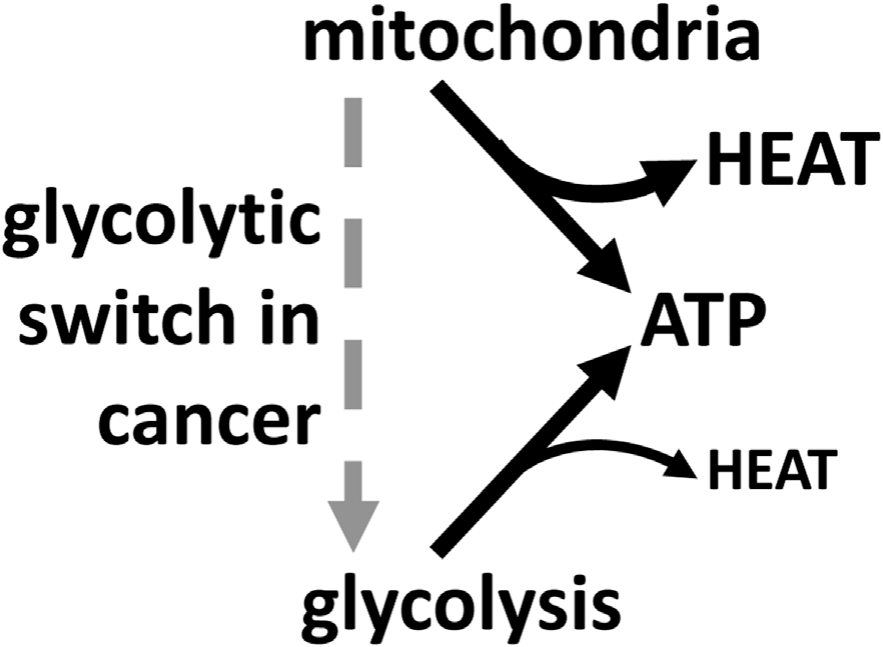
Mitochondria produce most of cellular heat, whereas glycolysis produces less heat per ATP synthesised. So, cancer cells may switch to glycolysis to produce less heat if and when tumours overheat.

In conclusion, glycolysis is efficient, and produces less heat per ATP than mitochondria, which might explain its preferential use in cancer cells and contracting muscle (Figure 6).

## Bioenergetic myth 5. Mitochondria are the main source of reactive oxygen species in cells

**FIGURE 7:**
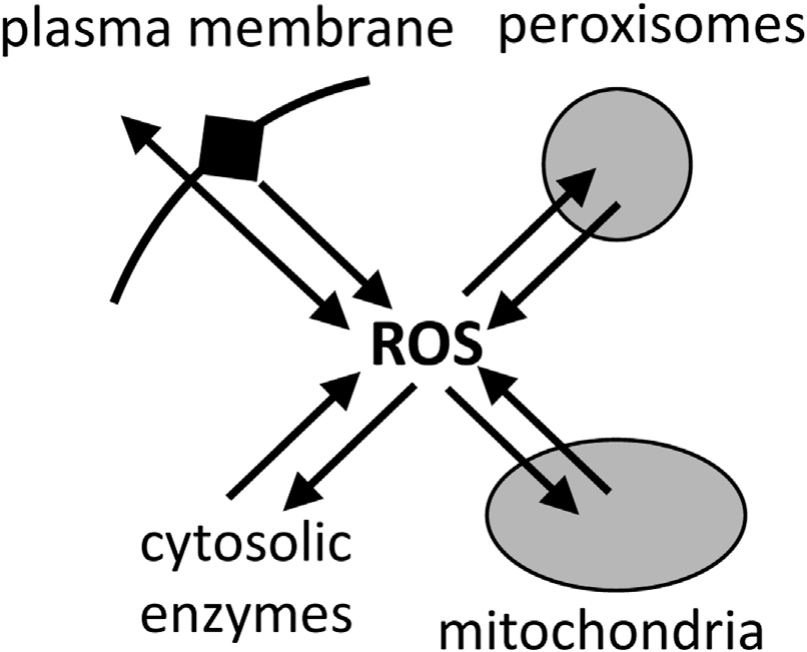
Reactive oxygen species (ROS) are produced mainly by plasma membrane, peroxisomes, mitochondria and cytosolic enzymes, but they are also rapidly consumed by these compartments, and the net fluxes in cells are unclear.

Mitochondria are generally assumed to be the main cellular source of reactive oxygen species (ROS), such as superoxide and hydrogen peroxide (Grivennikova and Vinogradov, 2013; Palma et al., 2024). However, there is no evidence that this is the case, and what evidence there is indicates that, in the cells and tissues that have been looked at, mitochondria are a significant source of ROS, but not the main source (Brown and Borutaite, 2012; Zhang and Wong, 2021). Peroxisomes, endoplasmic reticulum and plasma membrane each have a higher capacity to produce ROS than mitochondria, but the physiological rates in cells are hard to estimate (Brown and Borutaite, 2012; Zhang and Wong, 2021). Furthermore, mitochondria can consume superoxide and hydrogen peroxide at high rates (Mailer, 1990; Starkov et al., 2014), so it is unclear whether mitochondria are net sources or sinks of ROS in physiological conditions (Munro and Treberg, 2017) (Figure 7). Dey et al. (2016) found that mitochondria were net sinks for ROS in cells. However, recent assay of total ROS release from cells, then inhibiting or quenching ROS from different sources, concluded that mitochondria were a significant source of cellular ROS, but not the main source, contributing between 4% and 44% of total ROS production in the 8 different cell types examined (Zhang and Wong, 2021). NADPH oxidases were the main source of ROS in most cell types investigated (Zhang and Wong, 2021). Of course, the finding that mitochondria are not the main source of ROS in cells, does not mean that mitochondrial ROS are not important for physiology and diseases, but it does suggest that other sources may be more important.

## Discussion

Mitochondria are important sites of energy transduction and ROS production, but they are not the main sites in the cell. So, Bioenergetics needs to adopt a less mitocentric view if we are to fully understand energy and ROS fluxes in cells. However, mitochondria are the main source of heat production in the cell, and this is an underappreciated role of mitochondria, vital to the life of mammals and birds. But too much heat production can sometimes be a problem, and in such conditions glycolysis may be the solution (Horsman, 2006).

## Data Availability

The original contributions presented in the study are included in the article/Supplementary Material, further inquiries can be directed to the corresponding author.
