# Supplementary material for: Bioenergetic myths of energy transduction in eukaryotic cells
Source: Front Mol Biosci. 2024 Jun 17;11:1402910. doi: 10.3389/fmolb.2024.1402910 (PMC11215017; doi:10.3389/fmolb.2024.1402910)
Supplement: Supplementary file 1 [file DataSheet1.docx]

**Appendix 1. Some basic thermodynamics and definitions.**

The first law of thermodynamics states that energy cannot be created or destroyed, but it can be transformed from one form to another. Thus, in an isolated system (no exchange of matter or energy with the environment) the sum of all forms of energy is constant. The internal energy (U) of a system is just the sum of all forms of energy in that system, and this can change as a result of heat transfer (Q), matter transfer or work on or by the system (W).

The second law of thermodynamics states that the total entropy (of a system plus environment) always increases when something happens. The entropy (S) of a system can change as a result of heat transfer (Q), matter transfer or change in the form or distribution of matter or energy within the system.

The Gibbs free energy (G) is defined (at constant pressure p and temperature T) as = H - T.S, where H is the enthalpy (defined as U + p.V). For biological systems, ΔV is negligible, so ΔH ≈ ΔU. For a closed system (exchanging heat, but not matter, with the environment), and with no work being done on or by the system, the Gibbs free energy change ΔG = ΔH – T.ΔS, and ΔH = Q, where Q is the heat uptake by the system. In such a closed system, the entropy change of the environment (‘external’) due a change within the system (‘internal’) is entirely due to the heat transfer (Q) and is equal to -Q/T, which is equal to -ΔH/T. So:

ΔG = ΔH – T.ΔS = -T.ΔS_external_ -T.ΔS_internal_ ­= -T.ΔS_total_

Thus, according to the second law of thermodynamics above, the ΔG for any process is always negative (ΔG<0), i.e. free energy always decreases when something happens.

However, if a reaction C 🡪 D with a positive ΔG_CD_ is coupled via an enzyme or transporter to a driving reaction A 🡪 B with a negative ΔG_AB_, then the reaction can be driven forward if and only if: ΔG_AB_ + n.ΔG_CD_ < 0, i.e. -ΔG_AB_ > n.ΔG_CD_. i.e. the negative ΔG of the driving reaction must be greater the positive ΔG of the driven reaction. n is the reaction stoichiometry i.e. the number of molecules of C converted to D for each molecule of A converted to B.

The thermodynamic efficiency of this coupling process can be defined as: n.ΔG_CD_ / -ΔG_AB_

**Appendix 2. The contrasting architectures of metabolic pathways, energy transduction pathways and signalling pathways.**

There are three different types of pathways in cells for transferring three different types of things, with three different architectures:

****(1) Metabolic pathways transduce matter/molecules, and are linear (or branched) combinations of enzymes (and transporters).

(2) Energy transduction pathways transduce free energy, and consist of coupled cycles, with no matter transferred.

(3) Signal transduction pathways transduce signals/information, and consist of cycles between inactive (e.g. A) and active (A*) forms of proteins, where the active form activates the subsequent cycle via regulatory interactions (broken lines). No matter or energy is transferred along the pathway, but the cycles consume free energy.

**Appendix 3. The mitochondrial respiratory chain goes in the wrong direction.**

The protein complexes of the mitochondrial respiratory chain were named I, II, III and IV based on the concept of an electron transport chain with electrons starting at complex I or complex II and continuing via complex III and ending up at complex IV. However, the order of reactions is somewhat arbitrary, because the chain consists of coupled cycles, which could be considered to start with NADH consumption and end with H_2_O production, or alternatively start with O_2_ consumption and end with NAD^+^ production. Which is correct? Either or neither. Both processes are coupled together, and can not exist without the other. The direction is a convention. However, there are some heuristic advantages in considering the respiratory chain as running from oxygen consumption to carbon dioxide production (Fig 1A).

Figure 1A. The mitochondrial respiratory chain depicted in the opposite order to normal.

Why should it be in this order, rather than the traditional direction? Because: i) ‘respiration’ means oxygen consumption and carbon dioxide production, and cytochrome *c* oxidase (complex IV) starts this process and the decarboxylases finish it, and ii) cytochrome *c* oxidase is the only really irreversible step in the respiratory pathway, and energy transduction pathways traditional start with the largest energy change. Additionally, putting oxygen at the end of the respiratory chain leads to the myth that oxygen is just a sink/dump for electrons that have exhausted their energy. Whereas putting oxygen at the start of the respiratory chain and the origin of energy transduction in mammals acknowledges that the disequilibrium between the redox potential of oxygen and our reduced cells is what powers animal cells. The reduced molecules of food are often thought to be what powers us, but they have the same redox potential as our cells – food generally consists of other cells. It is the breathing in of oxygen with its dangerous high voltage (redox potential), very different from the negative redox potential of our cells, which drives the respiratory chain, that powers our cells.
